# Improving the impact of non-pharmaceutical interventions during COVID-19: examining the factors that influence engagement and the impact on individuals

**DOI:** 10.1186/s12879-020-05340-9

**Published:** 2020-08-17

**Authors:** Holly Seale, Clare E. F. Dyer, Ikram Abdi, Kazi M. Rahman, Yanni Sun, Mohammed O. Qureshi, Alexander Dowell-Day, Jonathon Sward, M. Saiful Islam

**Affiliations:** 1 School of Public Health and Community Medicine, Faculty of Medicine, University of New South Wales, Level 2, Samuels Building, Sydney, 2052 Australia; 2 North Coast Public Health Unit, New South Wales Health, Lismore, NSW Australia; 3 The University of Sydney, University Centre for Rural Health, Lismore, NSW Australia; 4 Centre for Population Health, New South Wales Health, Sydney, Australia; 5 Program on Emerging Infections, Infectious Diseases Division, icddr,b, Dhaka, Bangladesh

**Keywords:** Pandemic, COVID-19, Community, Non-pharmaceutical interventions, Acceptance, Masks, Behaviour quarantine

## Abstract

**Background:**

During an evolving outbreak or pandemic, non-pharmaceutical interventions (NPIs) including physical distancing, isolation, and mask use may flatten the peak in communities. However, these strategies rely on community understanding and motivation to engage to ensure appropriate compliance and impact. To support current activities for COVID-19, the objectives of this narrative review was to identify the key determinants impacting on engagement.

**Methods:**

An integrative narrative literature review focused on NPIs. We aimed to identify published peer-reviewed articles that focused on the general community (excluding healthcare workers), NPIs (including school closure, quarantine, isolation, physical distancing and hygiene behaviours), and factors/characteristics (including social, physical, psychological, capacity, motivation, economic and demographic) that impact on engagement.

**Results:**

The results revealed that there are a range of demographic, social and psychological factors underpinning engagement with quarantine, school closures, and personal protective behaviours. Aside from the factors impacting on acceptance and compliance, there are several key community concerns about their use that need to be addressed including the potential for economic consequences.

**Conclusion:**

It is important that we acknowledge that these strategies will have an impact on an individual and the community. By understanding the barriers, we can identify what strategies need to be adopted to motivate individuals and improve community compliance. Using a behavioural framework to plan interventions based on these key barriers, will also ensure countries implement appropriate and targeted responses.

## Background

Over the last 20 years a range of public health emergencies have resulted from the emergence or re-emergence of a pathogen. While the characteristics of each situation has been unique, there have also been underlying similarities. One of the main threads that runs through the history of these events is the need for community participation and engagement in the strategies proposed by governments. Since the first report about a novel strain of coronavirus, severe acute respiratory syndrome coronavirus 2 (SARS-CoV-2) emerging in December 2019 [1], governments around the world has acted rapidly to introduce a range of public health measures (referred to as non-pharmaceutical interventions, NPIs) aimed at reducing contact rates in the population and thereby reducing transmission of the virus. These interventions include the measures that individuals and communities can take to slow the spread of infection especially during a period when vaccines and medical treatments are not available [2]. NPIs include compulsory measures underwritten by public health orders, such as closures of various services and establishments, quarantine/isolation and restrictions on movement; and voluntary measures, supported by health promotion, such as disinfection of hands and surfaces, mask use working from home and maintaining a physical distance from other. In some settings these measures are voluntary, whereas in others they are now enforced (such as, via fines and/or jail time). Alongside the use of these measures, there is the critical need to test all people with suspected infection as quickly as possible, to promptly isolate cases, and trace and quarantine their contacts.

Experience from prior events has underscored that considerable cooperation is needed from the public to successfully implement these NPIs during an outbreak or pandemic. However, it is not always easy to predict what the public reaction to an unfamiliar event could be. While pandemic and/or emergency planning guidelines aim to promote compliance with these strategies, badly judged communication approaches can lead to members of the community becoming “complacent, panicked, or otherwise acting in unhelpful ways” [3], setting back or interfering with the ultimate aim of the control efforts. Previous commentaries on pandemic plans suggest that documents have glossed over or failed to account for how members of the public may respond in an infectious disease event [3]. In addition many of these documents are based on the assumption that engagement with the public does not need to happen prior to (an event) but strictly once things start to occur and at that point facts (what is the disease/how is it transmitted) and instructions should be applied only [3]. It perhaps not surprising that these emergency control guidelines and plans lack sufficient recognition of the enablers and barriers to effective public responses [4].

It is essential that we understand the factors that impact engagement with these mitigation strategies, to facilitate realistic expectations amongst the community and to devise appropriate communication strategies to promote acceptance and uptake. A 2010 review paper by Bish and Michie identified several demographic and psychological determinants associated with a higher probability of adopting protective behaviours during a pandemic [5]. Building upon this work and to support current activities for COVID-19, the objectives of this review were to not only identify the key determinants impacting on engagement with individual protective behaviours and NPIs but to also explore the impact of these strategies on the individual. Lastly, we aimed to map the key issues identified using a behaviour change framework so that we could identify relevant interventions to support and enhance community participation.

## Methods

### Search strategy

This study involved an integrative narrative review focused on NPIs. These interventions (previously referred to as community mitigation measures) are a diverse group of measures that people, and communities can take to slow the spread of infection. They include personal protective measures (e.g., respiratory etiquette, face covering use and hand washing), quarantine of exposed community members and isolation of cases, measures aimed at increasing physical distancing (e.g., school closures and dismissals, redesign of living and working places, and postponing or cancelling mass gatherings); and environmental measures (e.g., routine cleaning of frequently touched surfaces) [6]. In the literature, they can also be grouped as either preventive or avoidance behaviours. When considering the adoption of these strategies, it is recognized that each community is unique, and that the selection will vary based on the level of community transmission, characteristics of the community and their populations, and the local capacity to implement strategies.

In this article, we aimed to explore published peer-reviewed articles that focused on the general community (excluding healthcare workers), strategies (both preventive and avoidance focused) and factors/characteristics (including social, physical, psychological, capacity, economic, motivation and demographic) that impact on the effective implementation. The topics that are covered in this review were identified by the researchers as being potentially relevant to government and to individuals and relevant at the time of writing. The review of each topic is not exhaustive but rather a reflection into the related issues impact of their use, as well as the potential strategies to promote engagement. Lastly, an extensive discourse into the ethical implications of these strategies has not been included in this review, as previous publications have covered this, and we did not want to do discredit to this important topic. In alignment with previous authors, we acknowledge that ethics (and values) are key factors that needs to be considered but outside the scope of the current paper. Lastly, this paper does not attempt to comment on the rational for the recommendations, nor the evidence of effectiveness for the NPIs being utilised during this COVID-19 pandemic.

### Eligibility criteria

We searched publications in English on *Medline, PubMed, Scopus* and *Google Scholar* for the period between January 1, 2000 and to March 5, 2020, using a combination of search terms including ‘social or physical distancing’, ‘community mitigation’, ‘non-pharmaceutical interventions’, ‘community’, ‘general public’, ‘quarantine’, ‘social/university/childcare closure’, ‘hygiene’ and ‘pandemic or epidemic or individual disease names’. Additional, handsearching of articles bibliographies was undertaken. As this was a rapid review, we limited our screening to the first ten (10) pages for each term evaluated. At least two authors were responsible for identifying and reviewing extracted articles for each of the focus topics and for coming to a consensus on the findings included in this paper. The team of authors included a mix of academics and those working within local health departments so that we could ensure a mix of views and to support the extraction and adoption of relevant study findings. The team included members with experience of working in low, middle, and high-income countries. This review included quantitative (survey-based, observational studies) and qualitative (in-depth interviews, focus groups) studies which focused on the ‘community’ in general or focused on a discrete section of the community. We also included studies that were undertaken in response to the emergence of infectious disease events (SARS, MERS, and 2009 H1N1/A pandemic influenza) as well as studies published on COVID-19 (as of July 2020) and hypothetical pandemics (pre 2009).

### Screening and analysis plan

A data extraction form was developed by HS and applied to the identified studies so that there was a level of consistency in the approach. The data extraction form included publication details, study methodology, setting and location, population targeted, and identified factors and recommendations from the authors. We performed a content analysis of all the data, summarized under the focus areas and then compared the findings. Using the behaviour change framework developed by Susan Michie and colleagues as an analytical lens for this review, we then applied the three essential conditions – capability, opportunity, and motivation (COM-B system) – to the identified issues to outline suggested intervention functions that could be implemented to address these deficits. The Behaviour Change Wheel (BCW) is a guide to designing interventions using a theoretical approach [7]. To help research to identify the target behaviour, the following steps are followed: (1) using behavioural terms define the problem, ensuring that one is specific about the target population and the behaviour; (2) from a list of potential competing behaviours select the target behaviour; (3) specify the target behaviour in terms of who needs to do what, when, where, how often and with whom; and then (4) identify the factor that needs to change in order to achieve the desired behaviour by analysing interview data using the Theoretical Domains Framework (TDF) [8]. The TDF comprises 14 theoretical ‘domains’ representing a range of possible theory-based facilitators and barriers to behaviour change [9].

## Results

Across each of the sections that were reviewed for this narrative paper, 53 papers were identified as being relevant and information was extracted using the screening tool. Given the rapid nature of the work and the ongoing developments with the COVID-19 pandemic, supplementary papers were included that have been published since the beginning of this pandemic. Not all papers are referenced into the results section, as some findings have been replicated and supported across numerous studies.

It is critical to firstly acknowledge the key role that psychological and social factors play in underpinning an individual’s willingness and actual adoption of behaviours or practices outlined in the following sections [5]. As these factors or combinations of factors can influence adoption of preventive and avoidance behaviours, we are going to dedicate this first section to reflecting on them. Factors such as how an individual perceives their susceptibility to the infection (rather than their actual risk), whether they believe the infection to be severe if acquired, their perceptions towards the efficacy of the mitigation strategy and their perceptions toward their ability to conduct the activity (self-efficacy), can all contribute to how an individual will engage with a preventive behaviour [10]. Further discussion about these factors is included in the sections below. Additional analysis can be found in the work of Bish and Michie [5], Catherine So-Kum Tang [11] and Joseph T.F. Lau [12] and others. Work published during this current pandemic continues to support the role that perceptions towards risk and efficacy have on the adoption of behaviour [13, 14]. The following sections will focus on the individual strategies or interventions that have been applied during this COVID-19 pandemic.

### Personal protective measures

#### Mask use

In the studies reviewed, most authors have reported some level of ‘willingness’ to wear masks in the community, with higher levels of compliance (or willingness) reported amongst women, older adults (aged > 50 years), the highly educated (results are inconclusive in some studies), married individuals, and adults with poor self-rated health and/or home duties [15–21]. In regard to gender, it has been suggested that males may perceive the use of masks as being un- “manly”, and not use them due to variations in perceptions around health-related consequences [22]. However, not all studies have reported gender differences in relation to the use of masks, which may reflect the country of origin of the study (i.e., Australia and The Netherlands which do not have a culture of community mask use) [19, 23].

In 2014, Sim and colleagues published a review on the use of facemasks, drawing on the principles of the Health Belief Model [24]. They reported that individuals are more likely to wear facemasks due to the perceived susceptibility and perceived severity of being afflicted with life-threatening diseases. While perceived susceptibility was the most significant factor determining compliance, they also identified that perceived benefits of mask use did have significant effects on mask-wearing compliance as well. Focusing on the later, during the SARS outbreak, Tang et al. found that individuals were 1.4 times more likely to wear facemasks if they had strong beliefs in the effectiveness of wearing the products [21]. Beyond a belief in the efficacy of mask use, Lau et.al. reported that during the 2009 H1N1/A influenza pandemic, those who perceived that there was a ‘very high’ fatality rate associated with infection were more likely to wear facemasks regularly in public areas (OR 1.64, *p* < 0.01) [25]. Lastly, a lack of adequate knowledge about a disease may also hinder mask-wearing compliance. A survey from Taiwan identified that individuals were four times less likely to practice appropriate preventive behaviours, including mask-wearing if they lacked correct knowledge about the fatality rate of avian influenza [26]. Issues of access and affordability amongst vulnerable groups will also impact on mask use.

When it comes to cues to action, it has been suggested that corporate knowledge of outbreaks, social acceptance and perceived pressure from different sectors including employers, mass media, government and family can all play a role in influence compliance. Media blitz and public health promotion activities supported by government agencies provide cues to increase the public’s usage of facemasks. The survey conducted by Tang et al. (during SARS) reported that participants who were more aware of environmental cues (reminders from family members and/or government) were found to be 2.4 times more likely to wear facemasks [21]. In one study from Hong Kong, the authors reported that 95% of their survey participants believed that it was their “civic responsibility” to wear a mask in public places as frequently as possible and that they would wear a mask if they have any symptoms [18]. This is linked to the idea of social norms (or perceived social pressures) and highlights a difference between pre-pandemic practices between the East and West [11, 24].

#### Hand washing/hygiene

Hygiene promotion includes hand washing and sanitizing, respiratory etiquette and avoiding touching one’s face (mouth, eyes, or nose with unwashed hands). Studies have identified that compliance with hygiene strategies, especially hand washing, can be suboptimal and that the correct method may not be applied [27, 28]. Handwashing adherence depends upon complex behavioural considerations that are still being examined [29]. Much of the behaviour is automatic, habitual, cultural or determined by stimuli that are not open to conscious scrutiny [30]. For example, a mother might report that she washed her hands to avoid germs for the sake of offering a rational explanation, whilst her real motivation might be habitual i.e. the discomfort of sticky hands or their smell. Hygiene can be described as the need to be clean. If hands look dirty, then one is prompted to wash them (human emotion = disgust) [31] . The same can be said for cleaning a house (prompts: kitchen, toilet, surfaces look dirty) because it appears soiled. These actions may arise due to the presence of a disgust-evoking elicitor [32]. Beyond that, other factors that influence hand-hygiene behaviour include: (1) gender [27]; (2) habits which are acquired in childhood [33]; (3) social facilitation [34]; (4) modelling by people who may have perceived influence or social diffusers; and (5) environmental barriers, where lack of facilities or inconvenience prevent hand hygiene (affecting around 60% of the world’s population) [35].

#### Respiratory etiquette

The literature on this topic is far more limited, as studies tend to focus on hand hygiene and fail to comment on coughing, sneezing, and spitting [36]. One cross-sectional study conducted in Hong Kong during SARS found that being older, female, more educated and having higher risk perceptions and being anxious were linked to a greater chance of adopting precautionary measures, including covering their mouths [37]. In comparison, a study undertaken in Korea (post outbreak of MERS) [38], reported that only 50% of their surveyed participants (adults aged 20–69 years) understood what the term ‘respiratory hygiene’ meant and the mean score for self-reported compliance with respiratory hygiene/cough etiquette was 2.37 ± 0.42 out of a possible 4 [38]. In support of these findings is a notable study from New Zealand that actually recorded coughing and sneezing events during the 2009 influenza pandemic to monitor respiratory hygiene behaviours [36]. The authors found that around a quarter of the respiratory events (27.3%) were uncovered, and there was infrequent use of the responses recommended by health authorities (i.e., covering with a tissue or handkerchief at 3.4% and covering with elbow or arm at 1.3%). Whilst a small study, it does highlight that the message of covering with elbow or arm (in the absence of a tissue) was not having the desired behavioural impact in that context.

### Isolation and quarantine

In the past, compliance with quarantine has been linked to certain life circumstances such as educational status, work-related and financial concerns, family needs and the behaviours of others [39]. Studies have repeatedly showed that women are more likely than men to comply with isolation or quarantine requirements [39]. Age has been reported to be linked to compliance, with younger adults less likely to be compliant (alongside healthcare workers) [19]. Lastly, being married has also been reported to be linked to higher levels of compliance with quarantine orders during an outbreak of avian influenza [17]. Beyond the demographic parameters, a range of attitudinal factors have been associated with compliance with quarantine including perceived susceptibility, perceived efficacy and trust in the authority have all been found to be linked. Interestingly, perceived severity was not a reported factor in one study but it should be noted that ‘proportionality’ plays an important role in public’s compliance to the restrictive measures so probably can’t be ruled out [40, 41].

Isolation and quarantine can involve not only physical confinement, but also cognitive, affective, and spiritual isolation due to the limitations in the interactions with, respectively, health workers, relatives, and religious leaders. One of the main concerns is about the impact on job security and compromises in income (as per the school closure). This concern has been reported in all income brackets but particularly of concern to those who earn <$30,000 per year, those who would not be paid if away from work (self-employed/casual staff members/those who are unable work from home), those who lived in urban areas, those aged 18–30, and those who have only graduation from high school. Beyond the concerns about income, were those related to the ability to function while in quarantine with reference to access to groceries, healthcare services (including refilling medications) etc. As highlighted in one study, a concern existed about not being able to provide evidence of ‘sick leave’ because of not being able to attend the doctor. The last line of concern was about the capacity to comply with quarantine orders in households with large numbers of inhabitants (presence of extended family and visitors) in limited space. This concern was raised in an Australian study focused on the risk of pandemic influenza in Aboriginal communities [42]. These findings are also relevant to other settings in which the family unit is not limited to children and parents. In the same study, concerns were raised about the impact on the ability to attend funerals and important family gatherings. Having allowances such as these may be detrimental for the greater good but beneficial for the community. During SARS, Canadian residents (from three regions) who participated in focus group sessions suggested that without reciprocal arrangements (social and material support), individuals may resort to breaking quarantine, effectively being “forced to spread the disease” [41]. Participants stressed that, to create an environment for compliance and to justify the use of restrictive measures, measures must be proportional to the risk that is perceived by the public.

It has previously been reported during the SARS outbreak and the 2014 Ebola outbreak, that those under quarantine reported fear, loneliness, boredom, and anger, alongside feeling worried about the effects of quarantine and contagion on family members and friends [43]. Loss of intimacy and social contact, culminating in physical and psychological isolation has been documented [44]. Further details about these factors is included in the review by Brooks et al. [45]. Limited access to external resources which would normally provide comfort such as books, music, and toiletries also resulted in difficulty. Social stigmatization and loss of anonymity have also been linked to hospital-based quarantine [46]. Factors that amplify these factors including the duration of quarantine, the fears of infections, having inadequate supplies, and unclear information and communication [45].

### School closure (childcare, primary/secondary school)

Studies conducted during the H1N1/A 2009 influenza pandemic found that parents may lack the motivation or willingness to comply with measures because: (1) they may not believe that school closure will have any impact; (2) they may not understand the rationale for the action; (3) they may not feel that their children are at risk for the infection; and (4) they may not understand the requirements [5, 47]. Lack of awareness of the technical term for ‘school closure’ tended to be associated with “permitting out-of-home activities”, misunderstandings also led to some parents still sending children to school [48]. Students may also be unaware about the advice given to avoid social or physical contact was given to prevent themselves from infecting others [49].

Questions have been previously raised about the capacity to reduce community interaction between young people during a school closure period. Compensatory behaviour among school children has been referred to as the ‘compensation of contact’ (i.e. children continuing to meet outside of school settings) and is recognized to be a key determinant of the success of school closure interventions [50]. If the contact rates of students rise during school closure periods, then the impact of the strategy is reduced [50]. However, the task of keeping the contact patterns of children low can be challenging; for example, studies focused on out-of-home activities during periods of school closure for the 2009 H1N1 influenza pandemic, documented contact rates of 20.5% in Japan [51]; 34% [52] and 69% [53] in the USA; and 75% [54] in Australia. In the Australian survey, the 233 students reported more than 850 out-of-home activities over 7 days [54]. The Australian authors felt this was an underestimation. Attending sports games/ team practices and congregating in playgrounds were the most common activities reported. There were also stories of high school children gathering for a local inter-school dance, the same evening that school closures were enacted in one suburb of Australia [55]. Perhaps not surprisingly, younger children are more likely to leave the house during school closure [51].

#### Financial burden

Loss of income (economic strain) due to family members needing to take time off work to care for children was one of the key issues reported in previous outbreaks and pandemics [56, 57]. In some settings, parents may not have the ‘luxury’ of working from home, due to the types of employment. School closures would confront single-parent households with the challenge of balancing income generation and childcare [4]. The issue of job security also was a common thread in studies [58–60]. The subsequent impact on business and the wider economy (from parents being absent from the workforce) was also raised in the event of prolonged outbreaks/pandemics. However, these concerns were not echoed in all studies. For example, Effler et al. found that loss of wages and childcare issues were not very important in their survey of parents during the 2009 H1N1 influenza pandemic. This is surprising given that 50% of their parents reported missing work due to school closures and a third had to make special childcare arrangements [54].

#### Self-care

In regards to assistance with keeping children at home, a pre-pandemic survey of 1697 US adults reported that 64% would not need any assistance [61]. Of those who said they would need help, 50% would rely on family members, 11% on friends and neighbours, while 34% would look to outside agencies (including government agencies, church and community groups, or voluntary agencies). However, concerns have been raised about people being forced to make compromises such as relying on self-care (leaving a child in his own care or in the care of a sibling younger than the age of 13). Self-care has been found (in non-outbreak/pandemic situations) to expose children to three types of elevated risks: (1) injury; (2) suffering emotional or psychological harm; and (3) poor development because of poor choices of activities when in self-care [62]. The child may also have higher levels of anxiety because they are poorly equipped to care for themselves/others and to deal with daily life [62]. It could be theorized that children in self-care may also be more likely to engage in compensatory behaviours.

#### Impact on delivery of school-based resources

The impact of school closure on children’s nutritional health has also been highlighted by several studies [56, 63]. Closing schools in the event of a pandemic could leave children without access to these school-based nutrition programs. For example, in the US the National School Lunch Program (NSLP) and the School Breakfast Program (SBP) enable schools (US public and non-profit private schools and residential childcare institutions) to provide meals to children during the school day. Disrupted learning was also raised as a key issue during the 2009 H1N1/A influenza pandemic. While governments to date have been able to extend holiday periods in China and in other Asian cities to delay the return to school date, prolonged school closures may result in students having declining test scores. While home-school or distance-school has been suggested as ways to mitigate any impact, these strategies may not be feasible to all students if they lack access to computers/internet [63]. In Taiwan, elementary school teachers provided tutoring for children over the phone in 2009, while classes were suspended due to H1N1 influenza, to reduce the impact on the students. In addition, they also monitored the health of the students and encouraged them to comply with the recommendations including adherence with chemoprophylaxis. In that setting, the authors reported a high level of support from parents for the closure [64]. In the US survey previously mentioned, the vast majority would be willing to give school lessons at home, although 47% would need some assistance [61].

### Mapping barriers and facilitators linked to capability, opportunity, and motivation

The COM-B model is adapted from the validated and well-known Behaviour Change wheel, now widely used in public health contexts as a method for designing behaviour change interventions. Drawing on this model, the barriers identified during this narrative review were mapped across the three elements of capability, opportunity, and motivation and suggested interventions outlined in Table 1.

**Table 1:** Identified factors impacting engagement with strategies and possible interventions to consider

COMB	Factors impacting engagement physical distancing, hand hygiene, mask use and cough etiquette	Factors impacting engagement with quarantine, isolation, and school closure	Examples of interventions
Capability	Low knowledge/understanding about these NPI strategies in the community	Low knowledge about the strategies or the rational for their use	Communication efforts need to focus on outlining the rational for the use of the NPIs, as well practical knowledge of the ‘who, what, when, where’.
	Low willpower to follow through with interventions	Low understanding about what you are allowed (and not allowed) to do when complying with quarantine/isolation recommendations.	Establish an understanding about the different terms (i.e. isolation vs. quarantine, school closure vs. classroom shutdowns). Avoid using them interchangeably.
			Develop simple checklists to assist with complying with school closure, isolation, or quarantine
		Low willpower to stay in the house for quarantine period	
			Guidance to the community about mask use must include information about where to purchase masks, the recommendations around type/material/fit, donning/doffing instructions.
		Conflicting information about school closure from different sources	
			Designated community spokespersons to stimulate action (i.e. Key influencers: school leaders, church/community leaders, national advocacy peak body for people living with underlying health conditions etc)
Opportunity: Social and physical	Unable to access or purchase products (costs or availability)	Financial ramifications for taking time off work	Legislation/funding to support low paid workers having time off work due to illness, isolation/quarantine, and school closure requirements.
	Uncertainty around exemptions for mask use		
	Employed in a public-facing occupation (e.g. retail, transportation, or service roles) or within health, aged or community and childcare, that prevents physical distancing.	It is inconvenient to be at home for quarantine period days due to work and other commitments.	Ensure there are effective systems in place to keep in contact with those impacted by school closure, isolation, quarantine measures: SMS vs. emails vs. social media messages.
	Physical distancing recommendations do not account for housing arrangements, collectivist approaches to childcare and the cultural expectation of family members providing care for each other when sick.	Negative impact on school education	Restriction: Limited the number of masks that can be purchased by the community (while provide a clear rational). Redirect towards the use of cloth masks.
		Misinformation and rumours spread via social media and other networks	
			Promotion of hand hygiene in public by providing hand sanitation stations at public events and public transport stations. Providing masks to people who cannot afford to purchase products. Links charities that can sew/provide masks to groups in need.
	Information regarding NPIs does not adhere to recommendations around health literacy		
	Quality of the translated materials does not support knowledge/skills development.		
			Ensuring that when schools are closed, information is also communicated to local sports clubs and other out-of-school clubs.
	Peers are actively discouraging the use of masks		
			Identify key influencers (champions) to model personal hygiene strategies via mass media/social media campaigns (ambassadors). Community peers being promoted to encourage each other (promoting solidarity and altruism).
			Guidance to healthcare workers on how to talk to the public about mask and other hygiene measures.
Motivation	Perceptions around risk, severity and consequences are misaligned	Low levels of trust the information being given by local health authorities.	Information/Education: Simple, user friendly information on the strategies with balanced information about their impact and how to reduce the issues. Testing the emotional content to ensure there is no backfire effects.
	Perceive the NPI to have a low level of effectiveness.	Misconceptions about the need to comply with the process	
			Acknowledge to the community that it is ok to feel worried and that in the coming weeks/months you may be asked to comply with [insert strategy]. We need to start priming out community about the possible need for introducing physical distancing measures.
	Lacking society cues to act		
	Feeling anxious or stressed about complying with strategy	Concerns about the ability to access the necessary supplies needed during quarantine periods (including medications, food, and other necessities).	
	May only be motivated to wash hands when they are perceived to be ‘dirty’		
			Peer to peer motivation to promote engagement, highlight individual responsibility to contribute to collective goal.
	There is a gender/age response impacting on compliance with hygiene measures.		
		Concerns about loneliness, boredom, and social stigmatisation	Communicating about the positive impact on communities from high levels of engagement- emphasizing social norms around the practices, that they represent the right and socially responsible thing to do.
	Low levels of trust in government or health authorities		
		Financial ramifications for taking time off work	
			Poster/social media campaign using gain framing messages to influence feelings and actions (affect heuristic).
			Use personal stories about people being in quarantine/had children out of school and what strategies they used. Consider using tailored approaches to target men and younger adults.
			Gamifying hygiene rules at schools, workplaces, and homes
			Incentives for adolescents/young adults to adopt mask use. Consider the use of social media
			Care restructure- looking to trusted family members and friends to care for children. Taking it in turns to care for each other’s children if no other arrangements can be made. Encourage people to talk to their neighbours about their needs/contacts in the event of a quarantine period.
			Consider the use of social mobilisation contracts: “As a community member, I pledge to protect my friends and family by … ..’ (could also use reciprocity)

## Discussion

Willingness to adhere to NPIs may be influenced by the severity of illness observed in the community, relative to the need for income and the level of community, individual, and family disruption. Compliance reflects the interaction of a range of modifiable and nonmodifiable factors including the availability of resources, socioeconomic status, perceived consequences that could result, and their perceived level of personal and local community risk [65–67]. From the literature, a dominant theme that emerged is how to create an environment that supports and promotes adoption. Previously it has been suggested that we investigate the value of rewards or suasion rather than compulsion or punishment. To support this approach, the strategies that are implemented need to ensure that: (1) they are proportional to the threat; (2) compliant communities’ members are offered reciprocal arrangements; and (3) there is clear and transparent communication from key stakeholders.

Emotionally driven behaviours such as fear and threat also need to be considered. However, appealing to fear may be useful in some situations but not in others, as it may lead to defensive reactions [68]. Coupled with this, is the issue of optimism bias, which is a cognitive bias that causes someone to believe that they themselves are less likely to experience a negative event [69]. This bias is common and transcends gender, nationality, and age. Concerns about risk are often heightened during novel threats (especially for an infection that people have no physical awareness of) and in situations when people feel they have limited or no control. As acknowledged by Peter M. Sandman and Jody Lanard, it is common for people to feel anxious and potentially alarmed when faced with this unknown situation [70]. To get used to the new threat on the horizon, people may start acting like it is already present. For example, Sandman and Lanard postulated that a person may start wearing a mask on the subway as a form of emotional preparation for a feared future disease outbreak. This behaviour allows people to ‘emotionally rehearse’ the situation, as it is a functional response. It needs to be legitimized and requires acknowledgement that this is an unfamiliar space, while avoiding promotion of over-reactions. As suggested, “fearful people feel better, not worse, when their fear is legitimized; it is a relief” [70]. As suggested by Sandman, an unfamiliar situation should be made as familiar as possible, so that citizens begin to take the risks a little more for granted and that the outrage level will decrease [71]. A key issue that was raised by Bavel and colleagues was around the impact that continued exposure to negative framed messages can have on people [10]. As highlighted in their review, was the suggestion that the intense media focus on the number of people infected and dying may potentially sensitize people to neglect their own personal risk [10]. More work is needed around this issue and how message framing may impact.

Masks are currently being promoted as a means of source control i.e. reducing the risk of transmission by people who have been infected but are asymptomatic and contagious. The challenge with this, is that we do not currently understand whether members of the public fully understand this concept. In the echo chambers of social media, there are posts focused on ‘mask use for all’ and ‘wearing masks for others’, however we do not have a strong understanding of whether this sentiment transcends into the wider community. Social media misinformation and lack of well-designed education programs without community engagement can impact on compliance, acceptability and proper mask wearing. This issue merits further examination. Further issues which have been raised around the use of universal mask use during COVID-19 include what type of mask should be recommended (cloth mask or surgical mask), whether the use of masks will lead to panic buying or hoarding (and potentially supply chain issues/shortages for healthcare workers), whether children aged 2 years and over should be wearing masks (or face shields) and around how to optimize the usefulness of the products. To support and encourage uptake, it has been suggested that their use should be restricted to situations where they will confer a benefit. This includes times when a person is in enclosed spaces such as on public transport, at the shops or at places of worship, workplaces or at entertainment venues [72]. It could be theorized that by encouraging more targeted use of masks, this may assist with enhancing compliance. Community members may appreciate the heightened risk of exposure in situations where physical distancing is not feasible or where there is more chance of prolonged and close exposure to people and hence the rational for mask use. They may be able to tolerate shorter periods of masks use, despite concerns about the mask being uncomfortable. In communicating about the use of facemasks, efforts should be made to normalize the behaviour, to reduce the risk of discrimination or stigma targeted at those that are exempted from use (for physical or mental health reasons) and which continue to focus on the community being part of the solution. As suggested by Wang et al. public health messages to the public must include: the rational for wearing the mask, how to select the proper mask, how to dispose the mask and a reminder to continue with other prevention and control measures [73].

Strategies and interventions to promote hand hygiene are well studied in the healthcare setting, but when it comes to the promotion of community compliance, the literature is somewhat more limited. Finding interventions that successfully improve hand hygiene remains a challenge; studies conducted to date have failed to show any impact on hand hygiene behaviours between intervention and control groups [74]. Despite this, there has been key work around communication to promote hand washing/hygiene. One such study highlights that currently communication materials infrequently exhibit information consistent with theories of communication for behaviour change. Instead they rely on fear-based messaging, instead of using positive emotional cues or social norm appeals. In moving forward, it is suggested that hand washing/hygiene educational materials should adopt concepts from positive deviance [75]. In addition to hand hygiene, it is necessary to investigate strategies to promote cough etiquette. While there is agreeance that we should not be promoting people to sneeze or cough into their hands (unless they can directly wash them), the recommendation to cough or sneeze into ones elbow may not be a social norm in all countries and for some people is may not be habitual. Further mass media and the use of targeted social media campaigns may help to normalize this behaviour. Lastly, there has been some discussion around replacing one behaviour with another. For example, instead of touching your face, hold a pen or take a sip of water. If we are going to try and implement these sorts of messages, we must design these campaigns with the communities.

In situations where there are low levels of perceived risk, parents may lack the motivation to enforce their children to avoid social interactions. Compounding this are opportunity issues such as conflicting messages coming from schools and health departments or insufficient information coming to support parents to home-school. This is perhaps not surprising given the results from a literature review from Uscher-Pines et.al that reviewed pandemic policies and practices around school closures and found that there is limited literature on school practices to promote physical distancing, as well as limited incorporation of school practices to promote social distancing into state government guidance documents [76]. In future events, it is suggested that public health officials must clearly communicate with parents both why the intervention (school closure) is necessary, and what the benefits to the community will be. The same messages need to be delivered by the schools as they may be a trusted agent in some communities.

Some of the most important lessons about the use of quarantine come from its application in Liberia for the Ebola epidemic. As summarized in a paper by Umberto Pellecchia [77], one of the key issues was the lack of community engagement and low levels of ownership within the community in the decision making and implementation process. Ensuring that the communication about the situation (especially in the issue of school closure), is not simply subjugated by external agents. Use the local knowledge and practices of the community to create an environment of dialogue. He suggested that scheduled meetings or forums for awareness at neighbourhood or village levels could have greater impact than high- level meetings in which citizens only receive ‘echoes’ of information via mass media [77]. Lastly, he proposed that we need to work with local leaders (Principles, Religious leaders, Community leaders) as well as healthcare providers/public health practitioners, and communication specialists to enhance reaching local communities during times when their engagement with these strategies is needed. These local agents may be seen as more trustworthy and hence will be accepted as more credible and satisfactory [78].

For some of the issues identified in this review, a solution focused on supporting behaviour change is not straightforward. The key example here is the issue of job security and sick leave. The promotion of self-imposed home curfews assume that people have the luxury to take time off as they can access sick leave benefits or can work remotely. However, as reported in one US paper, 33.6 million US workers (esp. low wage and gig workers (i.e. food delivery people/ride share drivers) cannot access sick leave and so are faced with the dilemma of *‘seeing pay checks shrink, or going to work and creating health risks’.* This is a reality that would be played out in many low- and middle-income countries as well. The consequence of this situation is that presenteeism — attending work while ill — among private-sector employees without paid sick days may extend the duration of the outbreak in that sector. While this issue needs to be addressed federally or within states (by the changing of laws), a short term solution would be to encourage businesses to implement company policy that protects the jobs of people who are required to take time off (for school closures or due to being isolated).

Having reliable and trustworthy information is key in this situation for citizens to act upon and slow down the spread of COVID-19. The challenge is that health literacy levels (or the skills needed to obtain, understand, and process) vary across communities. As an impact of lower health literacy, people are less likely to access services or to understand the issues related to their health. During a pandemic, this may equate to mistrust in the recommendations and/or misunderstandings about risk, and lower levels of uptake of recommended interventions, including testing. Complicating this picture, is that in an exploding market of COVID19 facts and fiction, simply knowing about the risks (i.e. functional health literacy) is insufficient; individuals also need to be able to critically assess the information. It is critical that we ensure that communication approaches are tailored so they meet the needs of the target communities, in terms of messages and dissemination strategies. We need to increasingly draw on the known principles of communication [79]. One example could be to utilize personal stories into our communication strategies to normalize our thinking about these strategies. Patient stories are often used to promote best practices around quality and safety in healthcare, the issue is that this thinking has not been applied in our communication around community mitigation strategies for COVID-19. We also need to think carefully about how we use objectiveness and empathy, solidarity, and altruism to garner engagement.

This review was intended to be a rapid review of the literature to support the current actions being implemented worldwide. It differs from previous studies in that goes beyond the psychological determinants that impact on compliance to also look at other know factors that either impact on engagement or arise because of participation. It can serve as a benchmark to see how knowledge and recommendations have changed because of new evidence emerging during the COVID-19 pandemic. However, it has several limitations which need to be acknowledged. Firstly, it is not a systematic review, it does not include all the published literature on a topic. The selection and inclusion of findings may have been subject to unconscious bias. However, our team of investigators included people from both academic and health departments which may have reduced this issue. We purposefully chose not to comment on the quality of the papers within this review and chose not to limit ourselves to past pandemics but to capture the literature focused on both hypothetical and real situations. Both qualitative and quantitative studies were included to garner a rich understanding of each NPI strategy. As part of our reflection on these issues, we have ensured that we have included suggested readings to support people’s understanding.

## Conclusions

One of the key steps in this context is to ensure that we have community participation and co-design on the development of communication messages and practical materials to support those who are imposed upon to comply with these community strategies. It is also important to map the strategies or interventions to ensure that we are addressing the relevant key individual and contextual factors that have been identified in this study and others. Lastly, we need to ensure that we have a community voice in all the interventions that we plan. Working with the community to design new communication messages, community outreach initiatives and support documents will ensure that they have a higher level of authenticity and that they are received and adopted by those we need to effectively engage.

## Data Availability

The datasets used and/or analysed during the current study are available from the corresponding author on reasonable request.

## Abbreviation

NPIs: Non-pharmaceutical interventions
